# Role of the atypical chemoattractant receptor CRAM in regulating CCL19 induced CCR7 responses in B-cell chronic lymphocytic leukemia

**DOI:** 10.1186/1476-4598-9-297

**Published:** 2010-11-22

**Authors:** Julie Catusse, Marion Leick, Mareike Groch, David J Clark, Maike V Buchner, Katja Zirlik, Meike Burger

**Affiliations:** 1 University Clinic of Freiburg, Department of Hematology and Oncology, Hugstetterstr. 55, 79106, Freiburg, Germany; 2 Faculty of Infectious & Tropical Diseases, London School of Hygiene & Tropical Medicine, University of London, Keppel St, London WC1E 7HT, UK

## Abstract

**Background:**

The non-signalling chemokine receptors, including receptors DARC, D6 and CCX-CKR, have recently been shown to be involved in chemokine clearance and activity regulation. The human chemokine receptor CRAM (also known as HCR or CCRL2) is the most recently identified member of this atypical group. CRAM is expressed on B cells in a maturation-stage dependent manner and absent on T cells. We have recently shown that it competitively binds CCL19. CCL19 and its signalling receptor CCR7 are critical components involved in cell recruitment to secondary lymphoid organs and in maturation. B cell Chronic Lymphocytic Leukemia (B-CLL) is a low-grade lymphoma characterized by proliferative centres (or pseudofollicles). Proliferative centres develop due to abnormal cellular localisation and they are involved in the development of malignant cells. CCR7 is highly expressed on B cells from CLL patients and mediates migration towards its ligands CCL19 and CCL21, while CRAM expression and potential interferences with CCR7 are yet to be characterized.

**Results:**

In this study, we show that B cells from patients with B-CLL present highly variable degrees of CRAM expression in contrast to more consistently high levels of CCR7. We investigated the hypothesis that, similar to the atypical receptor DARC, CRAM can modulate chemokine availability and/or efficacy, resulting in the regulation of cellular activation. We found that a high level of CRAM expression was detrimental to efficient chemotaxis with CCL19. MAP-kinase phosphorylation and intracellular calcium release induced by CCL19 were also altered by CRAM expression. In addition, we demonstrate that CRAM-induced regulation of CCL19 signalling is maintained over time.

**Conclusions:**

We postulate that CRAM is a factor involved in the fine tuning/control of CCR7/CCL19 mediated responses. This regulation could be critical to the pivotal role of CCL19 induced formation of proliferation centres supporting the T/B cells encounter as well as disease progression in B-CLL.

## Background

B cell Chronic Lymphocytic Leukemia (CLL) is the most frequent adult low-grade lymphoproliferative disorder with a highly variable course, characterized by the accumulation of a specific subset of B cells in the bone marrow, blood, and lymphoid tissue. B-CLL patients typically present with proliferation centres or pseudofollicles in secondary lymphoid organs and are characteristic of CLL amongst all the other B-cell malignancies. They favour a microenvironment where dividing malignant cells are in contact with T-cells and cytokines that nurture the proliferation of malignant cells (for review [1]). Chemokines and their receptors are expected to be closely associated with the formation of these proliferative centres by directing cellular localisation and interactions.

Chemokines orchestrating leukocyte trafficking and localisation are required for cell maturation as well as immune functions. Leukocytes undergo several stages of migration from organs of production to blood stream and later throughout their maturation and active time. The chemokines CCL19 (formerly ELC, MIP3-ß) and CCL21 (SLC, 6Ckine), by binding to their receptor CCR7, play a role in regulating the homing of mature DCs, and subsets of T and B cells to lymph nodes. Close contact between cellular subsets within lymph nodes allows antigen presentation to naïve T cells that will eventually mature into different effector subtypes (reviewed in [2]). CCR7 stimulation by CCL19 or CCL21 has recently been shown to result in MAP-kinase phosphorylation and this is likely to be involved in CLL cell survival [3]. CCL19 and CCL21, although activating the same receptor, are distinct in several features. For example, CCL21 is structurally prone to high affinity for glycosaminoglycans (GAGs) due to a C-terminal basic tail [4], whereas CCL19 is one of the chemokines with the lowest affinity for GAGs known to date [5,6]. In addition, interactions with CCR7 have different cellular outcomes for each chemokine. Binding of CCL19 to CCR7 results in internalization and degradation of CCL19 and receptor desensitization. Conversely, after ligation of CCR7 by CCL21, the receptor remains stable at the cell surface and its signalling capacity is limited [7,8]. It has also been shown that while CCL21 is produced at its site of action by fibroblastic reticular cells of the T cell zone and HEVs (High Endothelial Venules), CCL19 expression is restricted to non-endothelial cells in the T cell zone of secondary lymphoid organs and thus needs to be translocated to the HEVs [9]. Besides binding to CCR7, CCL19 and CCL21 both bind with high affinity to another member of the atypical chemokine receptor family: CCX-CKR [10,11]. This scavenger receptor efficiently regulates CCL19/21 bioavailability by degradation. However, CCL19/21 both avoid regulation by the two best characterized atypical receptors D6 and the Duffy antigen receptor for chemokines (DARC). While D6 is an effective scavenger of many inflammatory chemokines [12,13], DARC has been shown to be a key player in the transcytosis of CCL2 and CXCL8 from their production site in the tissue to the luminal side of the endothelium [14].

We have recently shown that a third CCL19 binding chemokine receptor exists: CRAM (also known as HCR or CCRL2). Encoded by the gene *CCRL2*, it is expressed on B cells in a maturation-stage dependent manner [15]. Besides CCL19, CRAM also binds the inflammatory chemokine CCL5 (RANTES) and the adipokine chemerin, while CCL21 fails to efficiently displace radiolabelled-CCL19 binding [16,17]. Ligand interaction does not induce any classical signalling response; instead CRAM displays a constitutive and clathrin-dependent cycling activity, resulting in the internalization of CCL19. Based on these results and the fact that CRAM has a modified version of the usually highly conserved DRY motif in its sequence (a feature of non-signalling chemokine receptors) we previously presented that CRAM is a silent, atypical receptor.

In this study, we investigated the role of CRAM in B-CLL, analysing CCL19 induced activation and found that CRAM is an efficient modulator of CCR7-CCL19 cellular responses.

## Results

### CCR7 and CRAM expression on lymphocytes

**Figure 1:**
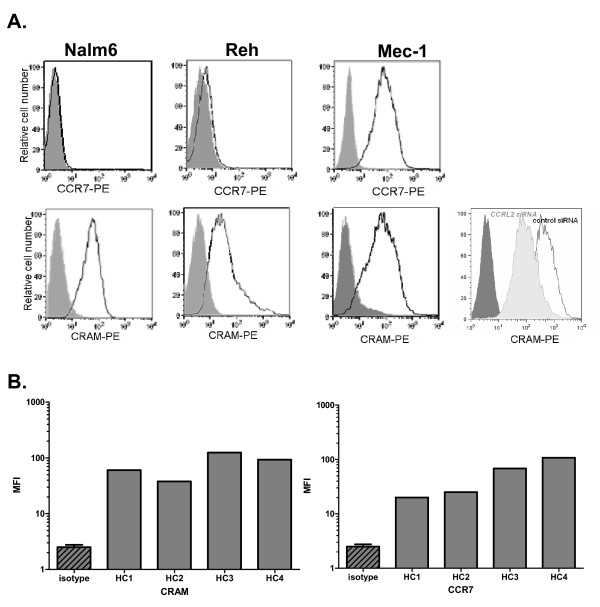
**FACS analysis of chemokine receptor expression on different cell types**. **A**. The histograms show the relative fluorescence intensity of the cells stained with anti-CRAM or anti-CCR7 antibody (black line) compared with the corresponding isotype control (tinted histogram). Nalm6 and Reh cells are positive for CRAM and negative for CCR7. MEC-1 cells are positive for both CRAM and CCR7. The additional histogram for MEC-1 cells shows the down-regulation of CRAM expression by siRNA transfection (light grey solid histogram). **B**. Mean fluorescence intensity (MFI) of CRAM and CCR7 of 4 healthy controls (HC). Isotype control has been repeated for each assay, the mean + SD of the four fluorescence geometric means is shown as a reference.

**Figure 2:**
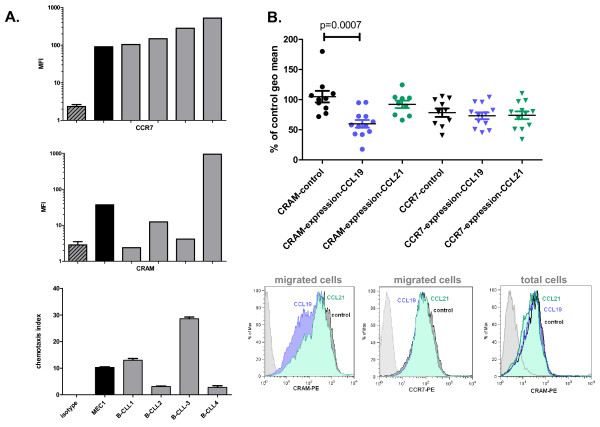
**Efficiency of CCL19 induced chemotaxis of B-CLL cells is independent of the expression level of CCR7 but not of CRAM**. **A**. The two upper panels depict CCR7 and CRAM expression at the surface of B cells from 4 patients with B-CLL (B-CLL1 to 4). Out of 23 patients tested, we selected two isolates of samples with similar levels of CCR7 and highly variable CRAM, and MEC-1 cells are shown as a reference. Results shown are MFI from a single flow cytometry experiment for the patients' samples; isotype control was repeated for each of them and represented as the mean of the 5 experiments + SD. The lowest panel depicts the mean + SD of chemotaxis toward 200 ng/ml CCL19 (one experiment done in triplicate). **B**. CCR7 and CRAM expression were tested at the surface of MEC-1 cells after migration induced by 200 ng/ml CCL19 or CCL21 and compared with the level of expression of cells before migration (control). A significantly lower CRAM expression is observed on cells that have migrated to CCL19 (unpaired T-test, p = 0.0007). The histograms below the graph depict one representative experiment, showing lowered CRAM expression in cells moving toward CCL19 (blue) but unaltered CRAM expression in similar experimental conditions with CCL21 (green). No alteration of CCR7 level of expression is observed (middle histogram). Incubation of MEC-1 cells with either CCL19 or CCL21 does not alter the detection of CRAM by the antibody used for the previous experiments (right histogram, one representative experiment).

In a previous publication we had shown CRAM to be highly expressed in pro- and pre-B primary cells as well as in corresponding cell lines [15]. The characterization of CRAM and CCR7 expression at the surface of cell lines and primary B cells from healthy donor controls was completed in the present study. MEC-1 (a B-CLL derived cell line) showed high expressions of both receptors whilst Nalm6 (pre-B Acute Lymphoblastic Leukemia derived cell line) and Reh (Acute Lymphoblastic Leukemia) cells had a similar level of CRAM but no CCR7 (Figure 1A). The expression of both CRAM and CCR7 at the surface of B cells from different healthy controls was shown to be consistent (Figure 1B and [18]). In parallel, the expression of CCR7 and CRAM was investigated at the surface of B cells from 23 CLL patients. Whilst CCR7 had a high but limited range of expression level (Figure 2A, data not shown and [3]), CRAM expression level was considerably more variable with regard to geometric mean fluorescence values, ranging from 2 to 949 (Figure 2A and data not shown).

### CRAM expression is associated with a reduction in CCL19 induced migration

The apparent inconsistency of CRAM expression in B-CLL led us to investigate the potential consequences for CCL19 induced migration. Two B-CLL primary cells samples paired for their similar levels of CCR7 yet variable levels of CRAM were selected (Figure 2A) and tested for their capacity to respond to CCL19 induced chemotaxis. A higher expression of CRAM was associated with a lower efficacy of chemotaxis (Figure 2A). To further investigate this observation, migrated cells from the lower wells of chemotaxis chambers, after migration towards CCL19 or CCL21, were stained for CRAM and CCR7 (Figure 2B). The CCR7 level of expression was not altered on the migrated cells. However, CCL19-induced migration was associated with a lowered expression of CRAM, not observed for CCL21. This observation is the result of cell selection rather than of receptor down-regulation as CCL19 does not induce CRAM internalization (CRAM constitutively recycles in a ligand independent manner [16]). Importantly, neither CCL19 nor CCL21 interfered with CRAM detection by the antibody used in the previous experiment (Figure 2B, lower right histogram). High levels of CRAM reduce the ability of cells to migrate toward CCL19, whereas it is not important for migration to CCL21 under the same conditions.

### Blocking CRAM potentiates responses to CCL19 but not to CCL21

**Figure 3:**
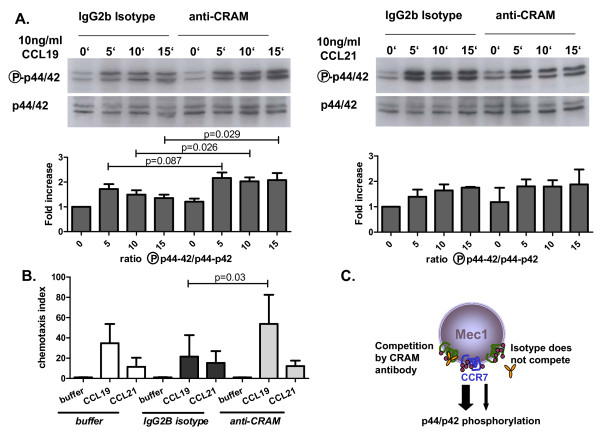
**CCR7 activation by CCL19 but not CCL21 is increased by anti-CRAM blocking antibody**. **A**. MEC-1 cells were incubated with 10 ng/ml CCL19 (left) or 10 ng/ml CCL21 (right) in the presence of an anti-CRAM antibody or of relevant isotype control for the indicated periods of time. Addition of anti-CRAM antibody increased the phosphorylation of MAPK p44/42. Upper panels show western blot results of phosphorylated or control p44/42, lower panels depict the quantification of five and three independent similar experiments for CCL19 and CCL21, respectively. The intensity of each phosphorylated p44/42 band has been normalised to the corresponding unstimulated control, and the result expressed in function of the time 0 point for isotype control for each experiment. Mean + SD are depicted, unpaired T-test analysis results in significant p values for 10 and 15 minute incubations with CCL19. No significance was observed with CCL21. **B**. The potentiation of CCL19 induced response by anti-CRAM antibody was confirmed in a chemotaxis experiment, where MEC-1 cells were stimulated to migrate toward CCL19 either in the presence of antibody against CRAM (light grey) or of corresponding isotype control (dark grey). CRAM antibody induced a significant increase of migration toward CCL19 (unpaired T-test, p = 0.03). No changes were observed for similar experiments with CCL21 (data from 3 independent experiments done in triplicate). **C**. Schematic illustration of molecular mechanisms explaining the previous results: potentiation of CCL19 induced p44/42 phosphorylation by CRAM blockage.

MEC-1 cells express both CRAM and the classical receptor for CCL19: CCR7 (also the classical receptor for CCL21) and were used to investigate MAPK phosphorylation after stimulation by CCL19 or CCL21 (Figure 3A). To evaluate the implications of CRAM expression on CCR7 activity, we blocked CRAM functions utilising different methods and analyzed the effects on CCL19 induced CCR7 activation. As phosphorylation of p44/42 has been shown to be involved in CCR7 induced B cell activation [19-21], we examined phosphorylation in the absence or presence of CRAM blocking antibodies. Addition of CRAM specific antibody potentiated the CCL19 triggered response. Furthermore, CRAM-antibody induces a longer signalling profile suggesting that CRAM could be involved in limiting CCR7 activation (Figure 3A). To assess the specificity of this observation, a similar experiment was conducted using CCL21 instead of CCL19. The antibody-induced potentiation of MAPK phosphorylation was not observed with this chemokine (Figure 3A). Similar results were obtained in chemotaxis assays (Figure 3B). The addition of the isotype control antibody did not alter the chemotactic profile of CCL19 or CCL21. However addition of antibodies against CRAM enhanced chemotaxis toward CCL19 but not toward CCL21.

**Figure 4:**
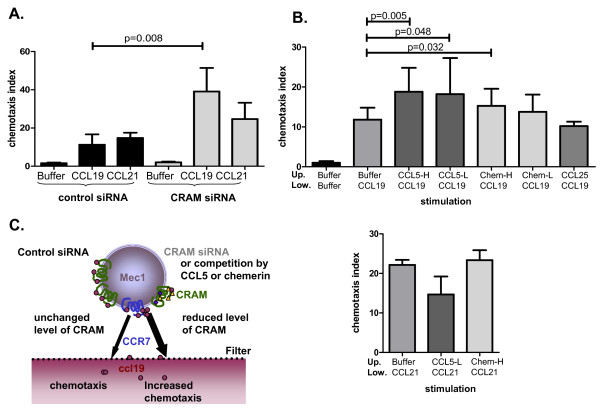
**Potentiation of CCL19, but not CCL21 induced chemotaxis by CRAM blockage**. **A**. Upper panel: MEC-1 cells (CRAM^+^/CCR7^+^) were used to do a chemotaxis assay toward 200 ng/ml CCL19 or CCL21, 48 h after transfection with negative control siRNA (black) or *CCRL2 *siRNA (grey). Experiment was carried out 5 times in triplicate, data shown are means + SD and statistical analysis were done by Mann-Whitney U-test, all chemotaxis indexes reached significance when compared with migration toward buffer (p ≤ 0.05): potentiation of CCL19, but not CCL21 induced chemotaxis by CRAM down-regulation. **B**. Upper panel: Chemotaxis migration toward CCL19 was done adding inert CRAM ligands in the upper part of the well CCL5 (H = 50 ng/ml, L = 5 ng/ml) and chemerin (H = 500 ng/ml, L = 50 ng/ml) or CCL25 as a control of exclusion for CCX-CKR involvement in the effect observed. Experiments were carried out 3 times in triplicate. Data shown are means + SD and analysis using Mann-Whitney U-test, all chemotaxis indexes reach significance when compared with migration toward buffer (P ≤ 0.001). Lower panel: as a CRAM specificity control, similar experiments were done, this time toward CCL21, for the conditions where significant changes were observed with CCL19. Neither CCL5-L nor chem-H altered the chemotaxis induced by CCL21. **C**. Schematic illustration of molecular mechanisms illustrating the previous results: potentiation of CCL19, but not CCL21 induced chemotaxis by CRAM blockage by competitors or reduced level of expression of the receptor.

**Figure 5:**
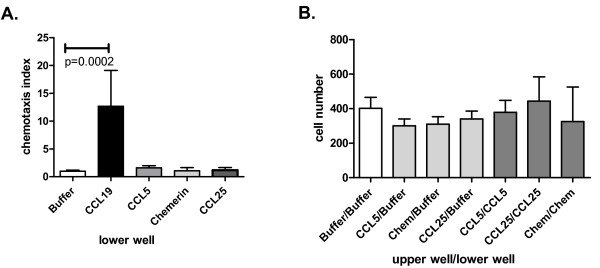
**MEC-1 mobility in presence of CRAM and CCX-CKR ligands**. **A**. CRAM ligands CCL5 (50 ng/ml) and chemerin (50 ng/ml) did not induce cellular migration by themselves; CCL25 (100 ng/ml), a control ligand to exclude CCX-CKR involvement in our observations, was also not able to induce MEC-1 chemotaxis, CCL19 (100 ng/ml) was used as a positive control (2 independent experiments done in triplicate. Data shown are means + SD, analysed using Mann-Whitney U-test). **B**. Absence of chemokinesis induction by the chemokines placed in the upper well or in upper and lower wells (one experiment done in triplicate).

To support these observations, experiments were conducted without blocking antibodies and in a different pathway of activation. CRAM expression was down-regulated by siRNA transfection in MEC-1 cells, and chemotaxis assays were performed using CCL19 or CCL21. siRNA achieved a CRAM down-regulation of approximately 30% (Figure 1A), low albeit sufficient to effect chemotaxis (Figure 4A). CRAM down-regulation resulted in enhanced CCL19 mediated chemotaxis but did not affect CCL21 induced chemotaxis of MEC-1 cells. This suggests that CRAM regulates CCL19 and thereby CCR7 activation, probably by competition either of ligand binding or by modifying/influencing activation. Finally, we obtained concurring results in an additional MEC-1 (CRAM^+^/CCR7^+^) chemotaxis assay. In this experiment, CRAM was blocked with its alternative ligands: chemerin and CCL5 (Figure 4B). Addition of CCL5 to the upper part of the chemotaxis well significantly increased the migration toward CCL19, yet this did not modify migration toward CCL21. This observation suggests that addition of CCL5 was not responsible *per se *for this increased migration but that there was a cooperative effect with CCL19. CCL5 addition was tested at two concentrations (CCL5-High: 50 ng/ml and CCL5-Low: 5 ng/ml) which showed significant decreases of chemotaxis (p = 0.005 and 0.048 respectively). Chemerin addition also resulted in significantly enhanced migration toward CCL19, but only the highest dose tested (Chem-High: 500 ng/ml) was statistically significant (p = 0.032). As low levels of RNA from classical receptors for chemerin and CCL25 (respectively ChemR23 and CCR9) and proteins from CCR5 and CCX-CKR were detected in MEC-1 cells (Additional file 1, Figure S1), we proceeded to conduct additional chemotaxis controls. CCL5 (for CCR5 expression), chemerin (for ChemR23) and CCL25 (for CCR9 and CCX-CKR) influences on the MEC-1 migration capacity were defined by assessing chemotaxis (Figure 5A) or chemokinesis (Figure 5B). None of these assays showed any conclusive involvement of CCL5, CCL25 or chemerin in MEC-1 migratory behaviour. No significant alteration of CCL19-induced migration was observed with CCL25, indicating that there was no involvement of CCX-CKR in our observations (Figure 4B). In addition, we controlled CRAM versus CCX-CKR involvement by reproducing our chemotaxis regulatory experiment with CCL21. CCL21 induced migration was not enhanced by the addition of other CRAM ligands (Figure 4B). Overall these experiments provide some insight into the nature of CRAM involvement in CCR7 regulation, implying that CRAM expression influences CCL19 activity and that this system is affected by other factors, including other cytokines (CCL5, chemerin).

### CRAM regulation over time

**Figure 6:**
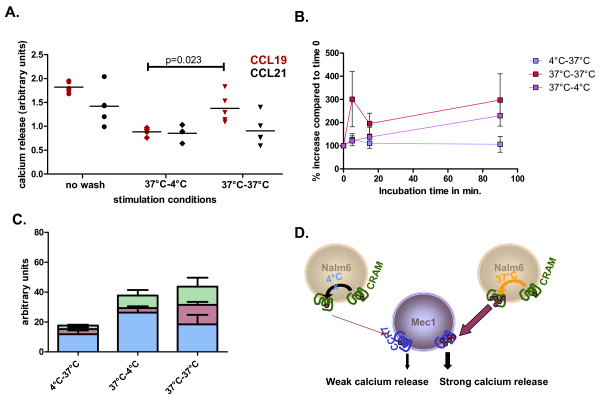
**Induction of calcium release by CRAM presented chemokines**. MEC-1 cells (CRAM^+^/CCR7^+^) were loaded with fluo-3/AM and then stimulated by Nalm6 cells (CRAM^+^/CCR7^-^) pre-incubated with CCL19 or CCL21 (100 ng/ml) for 30 min at 37°C and then treated as mentioned on the graph x axis. **A**. Calcium release was monitored by flow cytometry, allowing the discrimination between Nalm6 and MEC-1 cells, according to their SSC/FSC characteristics (Additional file 2, Figure S2). Spots represent results in arbitrary units from individual experiments and mean values of the five independent experiments are depicted as a line. Statistical analyses were done performing a T-test. **B**. Same experimental settings but this time changing the temperature of the initial incubation step (4°C: blue line, 37°C: pink and red lines), and the length of second incubation as indicated at various temperatures (4°C: pink line, 37°C blue and red lines). After incubations at different temperatures, the calcium release experiments were done at 37°C. **C**. Localisation of CCL19 in the cellular suspension used to induce the calcium release of MEC-1 cells was done using ELISA. Cells treated in the same way than for calcium experiments were used to quantify CCL19 in the supernatant (blue), at the cellular surface (pink) or internalised (green). When kept at 37°C throughout the experiments, the proportion of CCL19 internalized into the cells is higher, suggesting a possible time associated regulation of CCL19 by CRAM. **D**. Schematic illustration of molecular mechanisms explaining the previous results: incubation at 4°C blocks the internalisation of CRAM and hence its ability to sustain a CCL19 induced answer over time.

Finally, we examined CRAM modulation of CCL19 availability over time and performed a calcium-release assay. We stimulated MEC-1 (CRAM^+^/CCR7^+^) with Nalm6 (CRAM^+^/CCR7^-^) that had been pre-incubated with chemokines; Nalm6 cells do not express CCX-CKR, the previously described scavenger receptor for CCL19 (Additional file 1, Figure S1) hence CCL19 regulation observed cannot be attributed to it. Incubation of the Nalm6 cells (CRAM^+^/CCR7^-^) was done at 37°C to allow the internalisation of chemokines by CRAM and they were subsequently washed thoroughly with PBS buffer, before a further incubation (30 min) at 37°C or 4°C to allow or prevent respectively re-expression of potentially internalized chemokines. Chemokine incubated Nalm6 (CRAM^+^/CCR7^-^) were then added to MEC-1 cells (CRAM^+^/CCR7^+^) to investigate whether calcium release could be induced from chemokines presented at the cell surface of the Nalm6 cells (Figure 6A). Nalm6 cells that were incubated at 4°C post wash induced a smaller calcium release in MEC-1 cells, upon coincubation at 37°C, relative to control, unwashwed Nalm6 cells. This indicates that all the chemokines used in the first incubation step were not available for stimulation of MEC-1 cells (CRAM^+^/CCR7^+^) and therefore were not presented at the cell surface. MEC-1 cells co-incubated with Nalm6 cells, previously incubated at 37°C, had induced calcium release; almost as intense as before washing and significantly higher than stimulation by 4°C incubated Nalm6 cells. This implies that a proportion of CCL19, that was internalized in Nalm6 cells during the first incubation period, was available for MEC-1 stimulation during co-incubation in this experimental setting (Figure 6D). In comparison with CCL21, no difference was seen when Nalm6 cells were incubated at 37°C versus 4°C, supporting the involvement of a CCL19 specific receptor. Additionally, we monitored the CCL19-Nalm6 induced calcium release in MEC-1 cells after the two temperature conditions of initial exposure of the Nalm6 cells to the chemokine (4°C or 37°C) followed by a washing step and variable time and temperature conditions for the second incubation step (Figure 6B). This setting allows the monitoring of the internalization of CCL19 by Nalm6 (CRAM^+^/CCR7^-^) over time. When cells are initially incubated at 4°C, only surface bound CCL19 is present at the beginning of the experiment. Further incubation at 37°C did not change this observation, implying that the amount of chemokine associated with Nalm6 (CRAM^+^/CCR7^-^) and available for stimulation of MEC-1 (CRAM^+^/CCR7^+^) is sustained (blue trace). Nalm6 cells were incubated at 37°C prior to the washing step, to allow CCL19 to be internalized. This created two different pools of chemokine: one present at the cell surface and one intracellular. The latter would only be available to stimulate MEC-1 (CRAM^+^/CCR7^+^) when re-expressed at the cell surface and if CRAM is efficiently recycling rather than degrading CCL19; a 37°C dependent process. A correlation between incubation time and calcium release intensity was observed with enhanced responses when the second incubation period is done at 37°C. This resulted in CCL19 cell surface re-expression (purple and red traces). Quantification of CCL19 by ELISA was used to ascertain the location of the chemokines in suspension with Nalm6 cells after the longest incubation time (90 min). Three fractions were separated: supernatant, cell surface associated or internalized (Figure 6C). CCL19 associated to cells incubated first at 4°C and then at 37°C was found at lower concentrations than in the two other incubation conditions, with a very low proportion of CCL19 internalized and/or associated with the cells surfaces. CCL19 associated to the Nalm6 cells in the two other incubation conditions (preliminary incubation at 37°C followed by a washing step and further incubation at 4°C or 37°C) and presented higher concentrations of CCL19, with a similar quantity of CCL19 either associated to the cell surface or released in the supernatant (sum of blue and pink bars). However, when both primary and secondary incubations are at 37°C, the proportion of CCL19 in the cells or associated to the cell surface is increased, probably supporting signalling over time, an observation already seen in Figure 3. These data provide support to our hypothesis of CRAM involvement in active regulation of circulating CCL19.

## Discussion

CRAM has been described as a non-signalling receptor for CCL5 and CCL19 by our group and for chemerin by Zabel et al. [16,17]. This study investigated the implication of ligand binding to CRAM on cellular activity in the context of B-CLL. We demonstrate that CRAM expression is involved in the regulation of chemokine activity and we postulate that CRAM acts as a signal hub, capable not only of controlling circulating chemokines but also the activity of the corresponding classical chemokine receptors.

Following Comerford et al.'s hypothesis on the involvement of CCX-CKR in the alteration of circulating level of chemokine levels, we demonstrate here that CRAM modifies CCL19 availability and distribution and investigated the consequences of such scavenging on the functions of this chemokine [10]. Direct competition for CCL19 might be involved in this mechanism, but in light of the much higher affinity of CCL19 for CCR7 than for CRAM, additional inhibitory mechanisms are expected. For example, interference with efficient coupling of CCR7 to beta-arrestin is likely to be implicated. Arrestin involvement in CRAM internalization has indeed been shown by our group [16]. Arrestin-3 has also been shown to be involved in CCR7 internalization after stimulation by CCL19 but not by CCL21 [22]. The importance of arrestins in chemokine receptor internalization has been reviewed by Luttrell and Lefkowitz [23], and the different consequences of modified internalization on chemokine receptor activity have been summarized by Neel et al. [24]. The internalization of chemokine receptors is mainly seen as a desensitization mechanism and hence a way to interrupt and regulate signalling. However, for chemotaxis and calcium release, which are both mechanisms that need to be sustained over time, impaired internalization has been shown to be unfavourable. Our results indicate a regulation mechanism involving CCR7 receptor localisation at the cell surface, rather than internalization itself, at least as a first step. Variability in the CCR7 internalization scheme [7,8], as described for CCL19 and CCL21, could be a result of this preliminary modification of receptor localisation; however additional experimental evidence is required to confirm this hypothesis. When expressed on the surface of different cell types, CRAM might compete for binding of CCL19 with CCR7, but would then favour later CCL19 presentation to CCR7 expressing cells and cell surface accumulation, via CRAM, as suggested by our calcium release experiments (Figure 6).

When compared with other atypical receptor functions, CRAM's functions appear to be similar to DARC's, which has been shown to be a circulating-chemokine- "buffering" receptor [25] as well as a carrier of chemokines across endothelial cells. Thus far, we have assumed CRAM to be a narrow spectrum atypical chemokine receptor, similar to what has been published for CCX-CKR and its ligands: CCL19/21/25 [11,26]. It seems remarkable to have a chemokine regulated by two narrow spectrum atypical chemokine receptors when it is not regulated by the two large spectrum ones (D6 and DARC). The explanation for this observation is probably to be found in the central roles of CCL19 in immune cell maturation (T and B cells) and function (mature DCs). Two major differences exist between CCX-CKR and CRAM-mediated regulation of CCL19: first, the affinity of CCX-CKR for CCL19 is much higher than CRAMs. This does not necessarily reduce the importance of the role of CRAM in CCL19 regulation, as complementary roles for signalling chemokine receptors with different affinities has previously been described and documented (eg. CXCR1-CXCR2 [27]). The second major difference between these two atypical receptors is that CCX-CKR also regulates CCL21, the only other ligand for CCR7 [28], while we show in this article that CRAM regulation of CCL21 was weak or even absent. A differential role of CCL19 and CCL21 has already been shown by their activation of receptor CCR7: stimulation by CCL19, but not by CCL21, promotes CCR7 desensitization and CCL19 induces MAP-kinase phosphorylation much more efficiently in CCR7 expressing cells than CCL21, although both chemokines have similar affinities for CCR7.

CRAM expression has been shown to be broad [15,17,29], hence it could impact the chemokine regulation in various pathophysiological processes. So far, the most documented involvement of CRAM in pathology is its up-regulation on polymorphonuclear (PMN) cells during rheumatoid arthritis (RA) [29,30]. In this condition, the level of CCR7 expression is likewise up-regulated at the surface of T cells (but unchanged at the surface of PMN) and CCL19 secreted by mature DCs increased, emphasizing a thorough perturbation of CCL19 activities [29-31]. At the time, CRAM was shown to be up-regulated in RA, CCL19 was not known to be one of its ligands, and hence the impact of its expression had not been assessed directly. The modification of chemokine receptor pattern is a common feature of B cell-malignancies [32]. In B-CLL it appears that CCR7 activity alteration is associated with the formation of proliferative centres and lymphadenopathies [33]. We present here the first evidence for CRAM involvement in the regulation of B cell migration (Figure 2B, 3B and 4). In addition, we show that CRAM expression is also associated with a reduced MAPK activation (Figure 3) that is of particular interest when considering that chemokine induced MAPK phosphorylation is involved in cell survival in B-CLL. These two characteristics (modulation of migration and increased cell survival by CCL19) are essential for the creation and maintenance of proliferative centres in B-CLL and are supportive of the concept of CCR7 antagonism as a therapeutic target in B-CLL [34]. Furthermore, regulation of CCL19 by CRAM may be involved in a broader range of lymphoproliferative diseases. For example, a discrepancy between CCR7 level of expression and CCL19 induced chemotaxis has been shown for B cells in mantle cell lymphoma [18], CCR7 expression is altered in primary central nervous system lymphoma [35], and CCL19 is also associated with resistance to apoptosis in B-cell Acute Lymphocytic Leukemia [36]. In addition, CCR7 down-regulation is observed in T cell lymphomas when compared with healthy tissue [37]. We show *in vitro *that co-expression of CRAM interferes with the CCL19 induced activation of CCR7. Given the importance of CCR7 in secondary lymphoid organ organisation (for review [38]), its down-regulation, associated with an up-regulation of CRAM, is likely to be involved in T-cell accumulation in secondary lymphoid organs by spatio-temporal perturbation of cell localisation. Overall, our results provide a new perspective for the molecular mechanism supporting the development of these malignancies.

## Conclusions

We have presented new experimental data supporting the hypothesis of CRAM functioning as an atypical chemokine receptor involved in the regulation of CCL19 activity and as an integrative hub for different cytokines and receptors activities. We propose the hypothesis that CRAM functions as a regulator of altered cell migration in B-CLL.

## Methods

### Cell culture and reagents

All cell line media were supplemented with 10% Fœtal Calf Serum (FCS) and 1% penicillin/streptomycin. Nalm6 and Reh cells were maintained in RPMI and MEC-1 cells in IMDM in a humidified atmosphere (5% CO_2_) at 37°C (all from Invitrogen, Carlsbad, CA).

The murine monoclonal antibody against CRAM (anti-HCR/CRAM-A/B, Clone 152254) was produced by R&D Systems (Minneapolis, MN). The murine PE-labelled monoclonal antibodies against CCR7 (MAB197), CCR1 (FAB145P), CCR3 (FAB155P) and CCR5 (FAB182P) were purchased from R&D Systems. CCX-CKR (sc-46836) unlabelled antibody was purchased from Santa Cruz (Heidelberg, Germany). RPE-labelled rabbit anti-mouse IgG was from DakoCytomation (Glostrup, Denmark), Alexa-647 rabbit anti-goat (A21446) was from Invitrogen (Darmstadt, Germany) and chemokines were from R&D systems (Minneapolis, MN). PBMCs from Buffy coats of healthy donors were isolated by Ficoll density-gradient centrifugation. For isolation of chronic lymphocytic leukemia (CLL) cells, blood samples were collected from patients after informed consent. Peripheral blood mononuclear cells were isolated by density gradient centrifugation over Ficoll-Hypaque (Pharmacia, Uppsala, Sweden), and contained more than 90% CLL B cells [15].

### Flow cytometric detection of chemokine receptors expression

5 × 10^6 ^cells were incubated with the appropriate antibodies 30 min at 4°C in PBS, 0.5% BSA, and then washed in the same buffer prior to incubation with the secondary antibodies in the same conditions. After final washes, cell fluorescence was assessed using a FACScalibur counting 2 × 10^4 ^cells. Flow cytometry data were analysed with Flowjo.

### Western blot evaluation of p44/42 phosphorylation

Cells were starved in FCS free medium for 2 h and stimulated with chemokines for the indicated times at 37°C. Protein lysates were prepared with 100 μl lysis buffer (20 mM Tris/HCl pH 8.0, 150 mM KCl, 1 mM EDTA, 0.2 mM Na_3_VO_4_, 1% Triton X-100, 0.5 mM PMSF) with protein inhibitor cocktail (complete^®^, Roche Applied Science, Basel, Switzerland). Equal amounts of protein were separated by 10% SDS polyacrylamide gel electrophoresis and transferred onto PVDF membranes. Western blot analysis was performed using the appropriate antibodies recognizing the phosphorylated form of p44/p42 or pan-protein antibodies (#9101 and #9102, respectively) and were from Cell Signalling (Beverly, MA). Immunoreactive bands were visualized using horseradish peroxidase-conjugated secondary antibody and the enhanced chemiluminescence system (GE Amersham, Fairfield, CT). Densitometric quantification was done using Adobe Photoshop (Adobe Photoshop 9.0, Adobe, San Jose, CA, 2005). Data are presented as mean arbitrary density units normalized to the ratio between phosphorylated proteins to non-phosphorylated forms at time 0 in presence of the control isotype + SD. This statistical analysis as well as all the following ones were performed using Graphpad prism version 5.02.

### siRNA knocking down of CRAM expression

Expression of CRAM was knocked down using siRNA against the CRAM gene: *CCRL2 *(Santa Cruz, sc-77982, Santa Cruz) and siPORT lipid siRNA transfection reagent, following the manufacturer's instructions and using negative control siRNA when required (sc-37007).

### Chemotaxis

Chemotaxis experiments were done using NeuroProbe 96 well plate devices with 5 μm pore filter (Receptor Technologies, Warwickshire, UK). 50 μl of a 2 × 10^6 ^cell/ml suspension in HBSS, 0.1% BSA were loaded on the upper part of the filter after filling up the lower part with buffer (HBSS, 0.1% BSA) or chemokines in suspension in the same buffer. When stated, cellular suspension was completed with the indicated concentration of chemokines or chemerin. Migration was done over 1.5 hours and cell number evaluated by resuspending the migrated cells from the lower part of the well in 200 μl buffer and counting them by flow cytometry for a fixed period of time. The numbers of replicates and details of statistical analyses are described in the figure legends.

### Calcium release experiment

10^7^/ml MEC-1 cells were incubated for 45 min at 37°C in PBS buffer containing Fluo-3/AM probe at a final concentration of 2 μM (Invitrogen Molecular Probes), then washed and resuspended in calcium buffer (RPMI, 0.11 μM CaCl_2_, 0.1% BSA) to a final concentration of 2 × 10^6^cell/ml and incubated at 37°C for 5 min before proceeding to the calcium release assay at 37°C. The calcium release assay was performed by inducing MEC-1 with the addition of Nalm6 cells previously incubated with CCL19 or CCL21 (100 ng/ml) at 37°C (Figure 6A) or as indicated (Figure 6B). Nalm6 cells were then washed 3 times at 4°C in 5 ml PBS, 0.1%BSA, and incubated for a further 90 min in calcium buffer at 37°C (Figure 6A) or as indicated (Figure 6B) before being used to induce calcium release in MEC-1 cells. Results were monitored using a FACScalibur, FSC/SSC scattering allowing the differentiation between MEC-1 and Nalm6 cells according to their physical characteristics (Additional file 2, Figure S2).

### ELISA

Nalm6 cells were incubated in 500 μl of binding buffer (RPMI, 20 mM HEPES, 0.1%BSA) with or without 100 ng/ml CCL19 for 30 minutes at 37°C or 4°C as indicated. After this preliminary incubation step they were washed 3 times with 5 ml of PBS/0.1%BSA, and further incubated at 37°C or 4°C, as indicated, for 90 minutes in 500 μl of binding buffer. Three different sample collections were done: supernatant (1), cell associated fraction after a brief incubation of the cells with 50 μl of 10xPBS, to disrupt interactions between chemokines and their receptors (as described in [14]), and resuspension in a final volume of 500 μl PBS 1× for collection (2), finally the cells were lysed by 200 μl lysis buffer (20 mM Tris-HCl (pH 8.0), 150 mM KCl, 1 mM EDTA, 1 mM Na_3_VO_4_, 1 mM PMSF, 1% Triton X-100 and protease inhibitor mix) and resupended in a final volume of 500 μl with PBS (3). ELISAs were performed according to the manufacturer's instructions. Each fraction was normalised to its equivalent fractions collected in absence of CCL19.

### RNA-extraction and reverse transcription-PCR

As described in [15], total RNA was isolated using the RNeasy RNA isolation kit (Qiagen, Dusseldorf, Germany) according to the manufacturer's instructions. Residual DNA was removed by DNase I Digestion (Ambion, Austin, TX). The cDNA synthesis was performed using 1 μg RNA as a template for oligo-dT (12-18 mer) primers and 50 Units SuperScript II reverse transcriptase (Super Script first-strand synthesis system for reverse transcription (RT-) PCR; Invitrogen). The cDNA was amplified using Taq polymerase (Qiagen). The following primer pairs were used for PCR: CRAM-B: 5'-ATGGCCAATTACACGCTGGCACCAGAG-3' and the corresponding antisense primer 5'-CACTTCGGTGGAATGGTCAGGTTCTTCCCTC-3', GAPDH: the sense primer 5'-GGAGTCCACTGGCGTCTTCACC-3' and the antisense primer 5'-ATTGCTGATGATCTTGAGGCTGTTGTC-3' CCR9: 5'-GCCCAGGCCATGAGA-3' and the corresponding antisense primer 5'-AACCCACTGGGCCTGGCTGA-3' and ChemR23: the sense primer 5'-CCGGGACACAGCCAACCTGC-3' and the antisense primer 5'-CAGTGGCCAGGGGCAAACCC-3'. Annealing was done at 60°C.

## Competing interests

The authors declare that they have no competing interests.

## Authors' contributions

JC designed and performed the experiments described in figures 2, 3B and 3C, 4, 5 and figure S2 and was responsible for writing the manuscript. ML designed and performed the experiments described in figure 1A, 3A and figure S1 and participated in manuscript writing. MG performed the experiments described in Figure 1B. DJC critically reviewed the manuscript. MVB and KZ participated in experimental design and manuscript writing and MB provided guidance and supervised the work. All authors read and approved the final manuscript.

## Supplementary Material

Additional file 1 **Chemokine receptor expressions at the surface of the cells used in this article**. CRAM expression was controlled by RT-PCR on MEC-1, Nalm6, Reh and healthy control B cells. All cells were shown to be positive for CRAM. CCR9 and ChemR23 were also assessed for MEC-1 cells. Low expression levels were observed for both receptors. In the lower panel, protein expression was investigated for CCR1, CCR3, CCR5 and CCX-CKR in MEC-1 cells and CCX-CKR in Nalm6. A low expression of CCR5 and CCX-CKR was observed on MEC-1 cells. Click here for file

Additional file 2 **Physical differences allowing discrimination between Nalm6 and MEC-1 cells**. FSC and SSC settings allow direct discrimination by flow cytometry between Nalm6 cells and MEC-1 cells. Click here for file
