# Supplementary figures and images for: Role of the atypical chemoattractant receptor CRAM in regulating CCL19 induced CCR7 responses in B-cell chronic lymphocytic leukemia
Source: Mol Cancer. 2010 Nov 22;9:297. doi: 10.1186/1476-4598-9-297 (PMC2998479; doi:10.1186/1476-4598-9-297)

GAPDH CRAM CCR9 ChemR23

Mec1

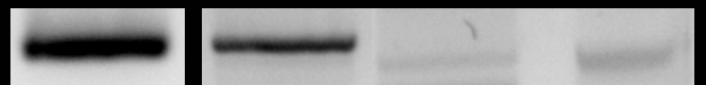

Nalm6

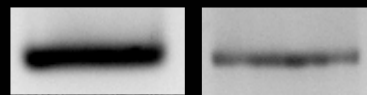

Reh

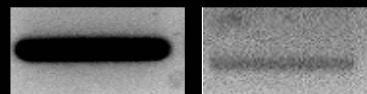

HC1

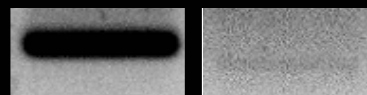

HC2

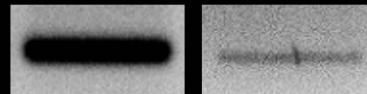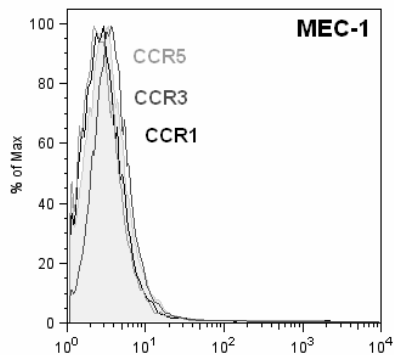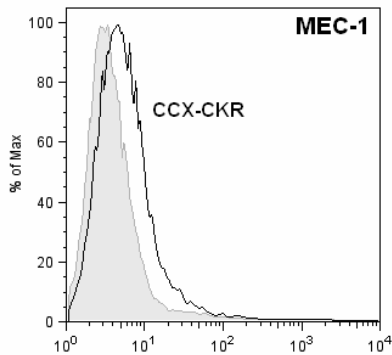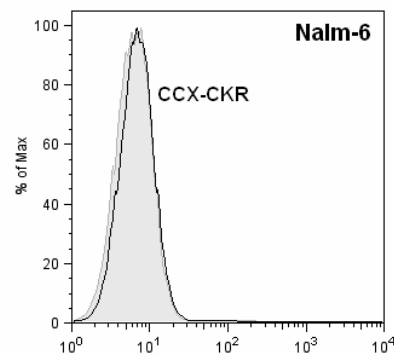

Supplement: Additional file 1 — Chemokine receptor expressions at the surface of the cells used in this article. CRAM expression was controlled by RT-PCR on MEC-1, Nalm6, Reh and healthy control B cells. All cells were shown to be positive for CRAM. CCR9 and ChemR23 were also assessed for MEC-1 cells. Low expression levels were observed for both receptors. In the lower panel, protein expression was investigated for CCR1, CCR3, CCR5 and CCX-CKR in MEC-1 cells and CCX-CKR in Nalm6. A low expression of CCR5 and CCX-CKR was observed on MEC-1 cells. [file 1476-4598-9-297-S1.PDF]

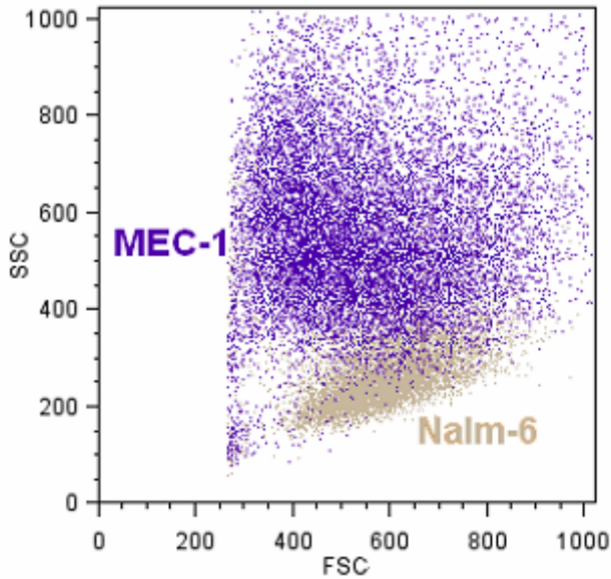

Supplement: Additional file 2 — Physical differences allowing discrimination between Nalm6 and MEC-1 cells. FSC and SSC settings allow direct discrimination by flow cytometry between Nalm6 cells and MEC-1 cells. [file 1476-4598-9-297-S2.PDF]
